# Impact of Proprioceptive Exercises on Pain, Balance, and Fall Risk in the Elderly With Knee Osteoarthritis: A Randomized Clinical Trial

**DOI:** 10.7759/cureus.70885

**Published:** 2024-10-05

**Authors:** Vahid Sobhani, S. Ebrahim Hashemi, S. Mohsen Mir, Arsalan Ghorbanpour

**Affiliations:** 1 Exercise Physiology Research Center, Life Style Institute, Baqiyatallah University of Medical Sciences, Tehran, IRN; 2 School of Rehabilitation Sciences, Tehran University of Medical Sciences, Tehran, IRN; 3 Student Research Committee, Baqiyatallah University of Medical Sciences, Tehran, IRN

**Keywords:** balance, elderly, knee osteoarthritis, pain, proprioceptive exercises, risk of falling

## Abstract

Background and objective: Osteoarthritis (OA), especially in the hips and knees, significantly impairs function and independence in older adults. Proprioceptive exercises have emerged as a beneficial intervention for managing knee OA. These exercises, which enhance proprioceptive feedback from mechanoreceptors in joints, muscles, tendons, and skin, are crucial for improving motor control and balance and have shown effectiveness in reducing symptoms despite limited evidence. This study aimed to examine the efficacy of proprioceptive exercises compared to conventional exercises in managing pain, balance, and fall risk among the elderly with knee OA.

Methods: A randomized, double-blind clinical trial was conducted on 54 elderly with knee OA. Participants were randomly assigned to either a proprioceptive exercise group (n=27) or a conventional exercise group (n=27). Outcome measures included pain (visual analog scale), balance (Berg balance scale), and fall risk (falls efficacy scale - international), which were assessed at three time points: baseline, six weeks, and eight weeks post-intervention.

Results: Both groups showed improvements in pain, balance, and fall risk (p<0.005). However, the proprioceptive exercise group exhibited significantly greater improvements in all outcome measures compared to the conventional exercise group (p<0.005).

Conclusions: Proprioceptive exercises demonstrated superior efficacy in reducing pain, enhancing balance, and mitigating fall risk in the elderly with knee OA. These findings suggest that proprioceptive exercises should be considered as a valuable component of comprehensive rehabilitation programs for this population.

## Introduction

According to the World Health Organization's 2020 report, the population aged over 60 is expected to double in developed countries over the next 30 years, consequently increasing both the retirement age and the strain on healthcare systems [1]. As individuals age, various bodily functions and characteristics change. While aging is a natural phenomenon, certain factors can expedite its manifestation, such as overall bodily weakness, heightened susceptibility to diseases, notable cognitive, mental, and functional alterations, as well as severe destructive changes in bodily tissues [1,2]. Osteoarthritis (OA), a degenerative joint disease, stands as the most prevalent form of arthritis and is recognized as a primary contributor to functional impairment and decreased independence among older individuals. Hip and knee OA emerge as significant sources of pain and physical disability among both adult and elderly populations. This condition significantly impacts the functionality of weight-bearing joints, particularly the hip and knee, often resulting in limitations in routine activities such as walking, transferring, and bathing. Additionally, OA poses considerable challenges in mobility, particularly in tasks such as climbing stairs and walking, making it the primary indication for total knee and hip replacements [3,4].

Due to the significant usage and stress endured by the knee joint, given its status as the largest synovial joint and a principal producer of joint synovial fluid in humans, it commonly becomes afflicted with painful conditions, notably OA [5]. While the articular cartilage lacks blood vessels and nerves, rendering it incapable of causing inflammation or pain independently, over time and through repeated exposure to inappropriate forces on non-cartilaginous components like the joint capsule, synovium, subcartilaginous bone, ligaments, and muscles, the knee joint undergoes various changes. These changes encompass bone regeneration, osteophyte formation, weakening of surrounding muscles, ligament laxity, and synovial effusion, all of which contribute to pain, discomfort, and disability [6,7].

Drawing upon an analysis of scientific literature, numerous medical and physical therapy interventions have emerged to alleviate OA symptoms, with a primary emphasis on exercise therapy [8-10]. Among these interventions, proprioceptive exercises stand out as a distinctive approach employed in knee OA management [11-15]. Proprioceptive signals originating from mechanoreceptors located in joints, muscles, tendons, and skin play a crucial role in the neural control of movement. The diminishment of proprioceptive input may disrupt muscle tone regulation, perturb postural reflexes, and consequently impact the spatial and temporal aspects of voluntary movements [11,12]. Movement triggers mechanoreceptors to provide proprioceptive feedback for both daily activities and more strenuous tasks. However, trauma and pathological conditions like OA can impede this feedback system, potentially heightening susceptibility to injury by compromising motor control [11,14,15]. Given the pivotal role of proprioception in motor control and balance during daily activities, it has been suggested that interventions aimed at rehabilitating motor function post-injury should prioritize proprioceptive training [11-15]. Smith et al. highlighted in their systematic review and meta-analysis that proprioceptive exercises demonstrate effectiveness in managing knee OA symptoms, albeit with limited evidence available [12].

Based on the literature review, there is a critical need to prioritize elderly individuals, specifically retired soldiers, who may experience knee OA, whether due to traumatic or non-traumatic causes associated with aging. Notably, there is a scarcity of research focused on those affected by knee OA resulting from injuries sustained during their service. Therefore, this study aims to examine the effects of proprioceptive exercises on pain, balance, and fall risk among the elderly with knee OA.

## Materials and methods

Study design and participants

This research was carried out as a randomized and double-blind clinical trial, overseen by a qualified physiotherapist. To ensure blinding, both the physiotherapist and the patients in the treatment group were blinded. The study obtained ethical approval under the code IR.BMSU.BAQ.REC.1401.094 from the Research Ethics Committees of Baqiyatallah University of Medical Sciences and was registered in the Iranian Registry of Clinical Trials with the registration number IRCT20190322043099N1. Participants were informed about the study's objectives and the treatment protocols. The study was conducted between May 1, 2023, and January 1, 2024.

Fifty-four elderly aged between 55 and 75 years, diagnosed with knee OA, were included in the study. Based on the balance variable reported in the Espejo-Antún study [15], a sample size of approximately 27 participants in each group was estimated using a confidence level of 95% and a power of 80%, calculated with the appropriate statistical formula.

Inclusion criteria required participants to have a BMI ranging from 25 to 30, clinical and radiological evidence of unilateral or bilateral knee OA, a physician's prescription recommending rehabilitation intervention as beneficial, symptom duration of more than three months, including pain, joint stiffness, functional impairment, reduced muscle strength, and limitations in daily activities. Participants were also required to have a numerical pain rating scale score between 1 and 5, and Kallgren and Lawrence grading scale (K/L) scores of 2 or 3. Exclusion criteria encompassed individuals with rheumatoid arthritis, polyarthritis, or systemic inflammatory arthritis history; those with mental impairments or using psychoactive medications affecting balance; patients with neurological conditions (e.g., stroke, cognitive disorders, cerebellar disorders, peripheral vascular diseases); individuals with ear disorders impacting balance, especially vertigo or vestibular dysfunction; history of knee, hip, or ankle surgery; recent or ongoing treatment with intra-articular knee joint injections; lower limb muscle strength below grade 3; and participants unwilling to continue with the study procedures [11,15-18].

Experimental procedures

In the initial phase of this study, a total of 54 patients were enrolled. The patients were randomly assigned to two groups using a closed envelope method, ensuring concealment of allocation by utilizing sequentially numbered, sealed, and opaque envelopes. On the first day of treatment, participants opened the envelope containing their group assignment. Consistent with prior research, both groups received instruction and training in a conventional physiotherapy exercise program for knee OA as outlined in a manual. Additionally, participants in the intervention group received instruction and training in a proprioceptive exercise protocol based on previous studies [14-22].

All exercises were conducted and supervised by a physiotherapist. Participants performed these exercises three times daily, with each session consisting of 15 repetitions, for six weeks, followed by a two-week follow-up period. The patients were allocated to two groups:

Group A, designated as the intervention group, received a combination of proprioceptive exercises and conventional therapeutic exercises. The proprioceptive exercises (n=27) included tasks such as one-leg balance, toe walking, heel walking, and advanced one-leg balance with closed eyes (referred to as blind advanced one-leg balance). Conversely, Group B, the control group (n=27), solely engaged in conventional therapeutic exercises. These exercises encompassed isometric quadriceps exercises, seated knee extension exercises, straight leg lifts, hamstring stretching exercises held for 20 seconds, performed in three sets with five repetitions each, and hip abduction and extension exercises.

All exercises were conducted and supervised by a physiotherapist. Participants engaged in these exercises three times daily, with each session comprising 15 repetitions over a period of six weeks, followed by a two-week follow-up period. The one-leg balance exercise involved standing on the injured leg in a fully relaxed hip and ankle position while bending the other leg from the knee for 30 seconds, followed by a rest period of 10 to 20 seconds, with three sets performed each day, consisting of 15 repetitions per set. For toe walking, participants were instructed to walk 20 meters on the toes of both feet, performing three sets per day with 15 repetitions per set. Heel walking exercises mirrored toe walking exercises, but participants were required to walk on their heels. The advanced one-leg balance exercise with closed eyes was akin to the one-leg balance exercise, with the added instruction for participants to close their eyes entirely during the exercise. This position was held for 30 seconds, followed by a rest period of 10 to 20 seconds, with three sets performed per day, each comprising 15 repetitions.

Regarding participant adherence to exercises, we monitored compliance through weekly check-ins and exercise logs completed by participants.

Measurements

The visual analog scale (VAS) is a graded scale ranging from 0 to 100 mm, used for patients' self-assessment of pain experienced in the past 24 hours. On this scale, 0 indicates no pain, while 100 mm represents the most severe pain imaginable. The VAS has shown strong validity and reliability, typically ranging from 0.85 to 0.95, in studies involving individuals with knee OA [23,24].

The falls efficacy scale - international (FES-I) was utilized to assess fall risk among elderly populations. This questionnaire comprises 16 items, each rated on a 4-point Likert scale (1 = no fear, 2 = slight fear, 3 = moderate fear, 4 = severe fear), resulting in a total score range of 16 to 64. The validity and reliability of the Persian version of the FES-I were previously established with a reported Cronbach's alpha of 0.98 [25-27].

The Berg balance scale (BBS) is a standardized assessment tool designed to measure the balance of elderly individuals through a series of 14 daily movement tasks. These tasks include sitting without support, standing with feet apart, standing with feet together, standing with eyes closed, standing with one foot in front of the other, standing on one leg, sitting down from a standing position, standing up from a seated position, transferring from bed to chair, turning 90 degrees to each side, completing a full 360-degree turn, picking up an object from the floor, reaching forward and transferring weight forward, and shifting weight between legs. The BBS demonstrates high levels of both internal and external validity, with reported coefficients typically ranging from 0.98 to 0.99 [28,29].

Statistical analysis

The statistical analysis was conducted using SPSS Statistics version 16 (SPSS Inc. Released 2007. SPSS for Windows, Version 16.0. Chicago, SPSS Inc.), with statistical significance defined as a p-value <0.05. The normality of the data was assessed using the Shapiro-Wilk test, confirming normal distributions for all variables. Descriptive statistics including ranges, means, and standard deviations were reported, and baseline characteristics between groups were compared using independent t-tests. To assess differences over time between the experimental and control groups, a 2 × 3 (group by time) repeated-measures ANOVA was performed. Effect sizes were calculated using guidelines from Fritz et al., where effect sizes of 0.25, 0.40, and >0.40 correspond to small, medium, and large effects, respectively [30]. Between-group comparisons were conducted using independent t-tests.

## Results

We enrolled 54 patients diagnosed with OA, with an average age of 63.15 ± 1.53 years and a mean BMI of 25.03 ± 1.90 kg/m². Table 1 displays the mean and standard deviation of demographic characteristics for all participants. There were no significant differences observed between the two groups in demographic variables, including age, height, weight, and duration of pain. Table 2 presents the results of a repeated-measures ANOVA examining differences in outcome measures (VAS, FES-I, BBS) between Group 1 and Group 2 across three time points.

**Table 1:** Physical characteristics of the participants (N=54) Values are presented as mean ± standard deviation. BMI: body mass index

Between-group comparison	Group B (N=27)	Group A (N=27)	Characteristics
0.86	63.11 ± 1.50	63.19 ± 1.59	Age (yr)
0.07	171.93 ± 3.43	169.48 ± 6.09	Height (cm)
0.53	73.07 ± 3.78	72.48 ± 3.19	Weight (Kg)
0.27	24.75 ± 1.61	25.32 ± 2.14	BMI (kg/m²)
0.49	3.85 ± 0.64	3.72 ± 0.73	Pain duration (yr)

**Table 2:** Repeated-measures ANOVA assessing for differences of VAS, FES-I, and BBS in two groups (n=54) Values are presented as mean ± standard deviation. ANOVA: analysis of variance, VAS: visual analog scale, FES-I: falls efficacy scale - international, BBS: Berg balance scale, ηp2: partial eta squared

Variable	Group A (N=27)			Group B (N=27)			Time (T) effect ηp2 (p-value)	Group (G) effect ηp2 (p-value)	T x G interaction ηp2 (p-value)
	Pre-test	Post-test	Follow-up	Pre-test	Post-test	Follow-up			
VAS	46.93 ± 2.92	35.74 ± 3.52	28.26 ± 3.20	46.33 ± 3.03	37.89 ± 3.27	30.04 ± 4.13	0.95 (p<0.001)	0.04 (0.13)	0.08 (0.03)
FES-I	37.15 ± 1.58	29.33 ± 2.25	23.33 ± 2.19	36.74 ± 1.70	32.93 ± 2.23	29.30 ± 2.18	0.94 (p<0.001)	0.51 (p<0.001)	0.59 (p<0.001)
BBS	35.22 ± 2.19	40.11 ± 2.22	42.07 ± 1.66	34.04 ± 3.06	35.56 ± 2.66	36.19 ± 2.30	0.86 (p<0.001)	0.43 (p<0.001)	0.63 (p<0.001)

Both groups showed significant improvements in VAS (pain intensity), FES-I (fall risk measurement), and BBS (balance measurement) after the intervention (p<0.005). Group A (proprioceptive exercises) exhibited larger mean differences and more substantial improvements in all assessed outcome measures than Group B (conventional exercises). The time effects were significant, indicating an overall improvement in three outcomes (p<0.005). Group effects were significant for FES-I and BBS. Also, the time-group interaction effects were significant for VAS, FES-I, and BBS (p<0.005), indicating that the change in these outcomes differed between the two groups.

## Discussion

Pain, impaired joint function (both gross and mechanoreceptor), and reduced quadriceps strength are primary contributors to balance disorders in knee OA [3-5]. The compensatory roles of hip abductor and ankle muscle strength in knee proprioception and balance highlight the potential impact of reduced proprioception on thigh muscle strength, mobility, and dynamic balance in OA patients. Moreover, joint pain can adversely affect muscle function (strength and activation) and sensory perception (proprioception and balance) [8-10,14,20]. Given these factors, we investigated the influence of proprioceptive exercises on pain, balance, and fall risk in elderly individuals with knee OA.

A variety of exercise interventions have been explored to enhance balance and proprioception in knee OA patients. Studies have reported benefits from exercises including walking, repetitive walking, closed kinetic chain activities, general movement, balance-specific training, aerobic exercise, tai chi, and aquatic exercises [11-15]. While most research has focused on individuals with mild to moderate OA, some studies have included patients with more advanced disease. To our knowledge, no previous studies have exclusively targeted elderly patients with this condition.

The main findings of this study were that proprioceptive exercises produced significant improvements in pain and balance measures, whereas a conventional exercise program produced non-significant improvements. It was suggested that proprioceptive exercises increase coordination between muscle groups and improve response to sensory information. In proprioceptive exercises, the patient progresses through exercises in different positions based on support and challenge their center of gravity. Thus, each exercise induces automatic and reflexive muscle stabilization that requires the patient to maintain postural control in various positions [12-16].

The knee OA is associated with reduced proprioception, muscle weakness, and pain [11-15], which can impair balance due to decreased sensory input from the knee joint. While the current study demonstrated improved balance and pain following proprioceptive exercises, likely due to enhanced afferent input and central processing, conventional exercise has also shown benefits in reducing pain, increasing strength, and improving proprioception and function [15-18]. However, our findings suggest that proprioceptive exercises alone may not suffice for optimal functional outcomes. To address this, exercises should prioritize neuromuscular control and daily activity demands. Theoretically, sensorimotor training, which enhances sensory input to the central nervous system, may be more effective than traditional exercise in improving knee joint sensorimotor function and proprioception [15-17]. Previous research supports the positive impact of motor and balance training on proprioception and function in OA patients [15-18,21].

The studied group showed a significant decrease in pain compared to the control group. In patients with chronic OA, the patient is usually caught in a closed-loop cycle called the physical remodeling cycle, in which the patient tries to compensate for the pain by creating an abnormal, restricted posture. This may lead to muscle spasms and reduced joint range of motion. This adaptation leads to increased pain [12]. The proprioceptive exercises were originally designed to manage chronic musculoskeletal pain conditions [12,14]. The superiority of proprioceptive exercises in addressing pain and balance deficits in knee OA likely stems from their ability to target multiple etiological factors concurrently. By challenging postural control and inducing reflexive muscle stabilization, these exercises may enhance the nervous system's capacity to process sensory information and generate appropriate motor responses. This improved sensorimotor integration can contribute to reduced pain perception as well as enhanced joint stability and functional performance [11-15,17]. Given the promising results of this study, it is recommended that proprioceptive exercises be incorporated into comprehensive rehabilitation programs for individuals with knee OA, especially in the elderly population. Future research should investigate the optimal dosage, frequency, and intensity of these exercises to maximize their benefits.

Limitations

The limitations of this study should be considered when interpreting the results. Firstly, the relatively small sample size of 54 participants may affect the generalizability of the findings to a broader population with knee OA. Additionally, the short follow-up duration limits the ability to assess the long-term effects of the intervention on pain intensity and motor control. While the study was double-blind, patients with prior rehabilitation experience may have recognized the differences between conventional and proprioceptive exercises, potentially introducing performance bias. Lastly, the lack of objective balance measures, such as force plates, limits the precision in assessing the impact of proprioceptive exercises on balance and motor control. Future studies with larger sample sizes, longer follow-up periods, and more objective assessment tools are recommended to confirm these findings and explore their broader applicability.

## Conclusions

Incorporating proprioceptive exercises into conventional strengthening and stretching programs for knee OA significantly enhances neuromuscular control, restores balance, and reduces pain intensity. Our findings demonstrate that proprioceptive exercises lead to more substantial improvements in functional outcomes compared to conventional exercises alone, particularly in terms of pain relief, balance, and fall risk. Given the strong relationship between pain and balance in knee OA, addressing both aspects through a combined exercise approach can offer a more comprehensive rehabilitation strategy. These results underscore the importance of including proprioceptive training in knee OA rehabilitation to optimize patient outcomes. Future research should explore the long-term effects of this intervention and its applicability to a broader population.
